# Non-invasive monitoring of cardiac function through Ballistocardiogram: an algorithm integrating short-time Fourier transform and ensemble empirical mode decomposition

**DOI:** 10.3389/fphys.2023.1201722

**Published:** 2023-08-17

**Authors:** Jingda Feng, WeiFen Huang, Jin Jiang, Yanlei Wang, Xiang Zhang, Qijie Li, Xuejun Jiao

**Affiliations:** ^1^ Department of Aerospace Science and Technology, Space Engineering University, Beijing, China; ^2^ China Astronaut Research and Training Center, Beijing, China; ^3^ National Key Laboratory of Human Factors Engineering, China Astronaut Research and Training Center, Beijing, China

**Keywords:** Ballistocardiogram, EEMD, STFT, non-invasive, morphological features

## Abstract

The Ballistocardiogram (BCG) is a vibration signal that is generated by the displacement of the entire body due to the injection of blood during each heartbeat. It has been extensively utilized to monitor heart rate. The morphological features of the BCG signal serve as effective indicators for the identification of atrial fibrillation and heart failure, holding great significance for BCG signal analysis. The IJK-complex identification allows for the estimation of inter-beat intervals (IBI) and enables a more detailed analysis of BCG amplitude and interval waves. This study presents a novel algorithm for identifying the IJK-complex in BCG signals, which is an improvement over most existing algorithms that only perform IBI estimation. The proposed algorithm employs a short-time Fourier transform and summation across frequencies to initially estimate the occurrence of the J wave using peak finding, followed by Ensemble Empirical Mode Decomposition and a regional search to precisely identify the J wave. The algorithm’s ability to detect the morphological features of BCG signals and estimate heart rates was validated through experiments conducted on 10 healthy subjects and 2 patients with coronary heart disease. In comparison to commonly used methods, the presented scheme ensures accurate heart rate estimation and exhibits superior capability in detecting BCG morphological features. This advancement holds significant value for future applications involving BCG signals.

## 1 Introduction

Monitoring cardiac activity has been a popular area of research in biomedical practices. However, current clinical monitoring methods, such as electrocardiography (Camps et al., 2021) and echocardiography (Ostvik et al., 2021), require specialized equipment and professional operations. Moreover, the electrodes or probes used can cause physical discomfort and psychological stress, particularly with long-term use (Sredniawa et al., 2020). As demand for non-invasive home health monitoring grows, BCG has emerged as a promising alternative capable of effectively assessing cardiovascular and respiratory activity (Inan et al., 2015). Recent advances in electronics and sensor technologies have led to a variety of ways to collect BCG signals. Sensors can be embedded in objects such as bed legs (Mitsukura et al., 2020; Jung et al., 2021), mattresses (Hwang et al., 2014), chairs (Rajala et al., 2018), pillows (Sadek et al., 2017), weight scales (Liu et al., 2022), and even a camera (Li et al., 2020) to enable monitoring in a non-sensory state. By eliminating the need for direct contact between the subject and sensors, these methods minimize physical and psychological discomfort. For example, sensors integrated into a hospital bed or home environment enable all-night BCG signal acquisition and effective heart rate detection, and non-invasive sleep monitoring (Nurmi et al., 2016).

Ballistocardiogram (BCG), a body vibration caused by heart beating, was initially discovered by Gordon in 1877. In 1936, Starr et al. provided evidence that the sensitivity characteristic of BCG can reflect muscle contraction non-invasively, which indirectly indicates the state of cardiac activity (Starr et al., 1978). BCG signals record the synchronous movement of the human body caused by left atrial blood pumping and indirectly reflect the mechanical action of the heart. Normal BCG displays consistency in a series of heartbeats and has repeatability, consisting primarily of H, I, J, K, and M, N waves. The largest amplitude in BCG signals is composed of H, I, J, and K waves, which can be illustrated by the letter W in shape.

Apart from heart rate, BCG signals are a valuable source of information that can effectively reflect cardiac function (Chang et al., 2021; Shin et al., 2021b). Among the BCG signal characteristics, the I-J amplitude is closely linked to changes in cardiovascular parameters. Studies have shown that the I-peak of the BCG reflects the maximum acceleration of ascending aortic blood, which can serve as a measurement of the mechanical forces of the heart (Guidoboni et al., 2019). Moreover, the J-peak of the BCG provides insight into heart blood ejection. The time interval between the R wave of the ECG and the BCG I and J waves can be used to analyze the time between cardiac contraction and aortic ejection. Positive correlations were observed between I-J amplitude and cardiac output (CO) as well as stroke volume, and also between I-J slope and heart rate (HR) (Rooij et al., 2015). Additionally, significant differences in the I-J interval, J-K interval, and wave group power between healthy individuals and patients with coronary heart disease (CHD) have been noted (Etemadi and Inan, 2018). Heart valve dysfunction impacts cardiac ejection and reduces the amplitude of I and J waves (Gubner et al., 1953). Several studies have indicated that the K waves of the BCG are often reduced or absent in most cases of aortic coarctation (Jiang et al., 2021a). These morphological features can only be detected by accurately identifying the IJK-complex. Once the IJK-complex is detected, the amplitude and interval of other BCG waves can be analyzed in greater detail.

The advancement of digital signal processing technology has provided powerful tools for processing BCG signals. Various signal processing methods have been applied to the processing of BCG signals. These methods include peak detector (Brink et al., 2006), template matching (Rooij et al., 2015), auto-correlation function (1 et al., 2013), cepstrum analysis (Bruser et al., 2015), fast Fourier transform, wavelet analysis (Paalasmaa et al., 2015), and empirical mode decomposition (Seok et al., 2021). In recent years, with the development of artificial intelligence technology, clustering-based (Paalasmaa et al., 2015) and deep learning methods (Cathelain et al., 2020; Hersek et al., 2020) have also been applied to analyze BCG signals. Additionally, multi-channel signal acquisition systems and multi-channel data fusion algorithms have been developed to enhance the detection system’s performance (Jung et al., 2021). Notably, the signal processing of each channel of a multi-channel system is based on a single-channel algorithm.

Most of the above algorithms were primarily designed to estimate IBI and heart rate, rather than accurately locating the position of a specific waveform in the BCG. These methods convert BCG signals into various correlation or wavelet coefficient curves, which may sacrifice BCG waveform information while providing relatively accurate IBI data. Locating the IJK-complex in the BCG signal without referencing another signal is a challenging task. This is because the waveform of the BCG signal shows substantial variability from subject to subject, and even the same subject shows significant variations at different periods or in different acquisition positions (Ibrahim, 2018). Generally, the HIJKL complex with a “W” shape is the most amplified and reproducible segment of the BCG signals, and the largest amplitude is typically found in the J wave. However, in certain cases, the amplitude of the H wave or L wave may surpass that of the J wave. Due to noise and the variability of the BCG signal waveform, the IBI estimation algorithm based on J wave detection often produces larger errors than those based on template matching and correlation function methods.

To date, a reliable and practical method to accurately locate the position of IJK waves in the BCG signal without referencing other signals remains elusive. This study proposes a novel BCG signal detection method based on the short-time Fourier transform (STFT) and ensemble empirical mode decomposition (EEMD), with the goal of identifying the position of the IJK wave in each BCG signal without relying on ECG and other signals. By relying solely on the BCG signal, this method can acquire heart rate, HRV, I-J amplitude, I-J-K interval, and other cardiac functional information. The performance of the algorithm was evaluated in 12 subjects.

## 2 Materials and equipment

### 2.1 Signals acquisition equipment

In the BCG signal acquisition circuit design, an electromechanical film (EMFi) sensor (Satu and Jukka, 2012) was used for BCG signal acquisition. The tiny pressure signal generated by breathing or heartbeat can induce charge changes on the corresponding surface electrode layer of the sensor. The high sensitivity and high input impedance characteristics of the EMFi sensor suitably facilitate the weak physiological signal acquisition. The BCG signal conditioning circuit comprises a charge amplifier, low-pass filter, signal separation, and secondary amplifier circuits. A charge amplification circuit based on OPA4340 (Texas Instruments, 2016) transformed the output signal of the electromechanical film sensor into a voltage signal. To mitigate high-frequency noise interference, a second-order Butterworth low-pass filter with a cut-off frequency of 30 Hz was employed.

In the supine position, the respiratory signal’s amplitude exceeds that of the BCG signal. Due to the close frequency band range of the respiratory (0.2–0.5 Hz) and BCG signals (1–12 Hz) (Ibrahim, 2018), filtering the respiratory signal through an analog filter is challenging. Thus, to suppress respiratory signals’ narrow frequency band and high amplitude characteristics, a dual T-Notch filter with a 0.3 Hz center frequency was designed. The dual T-Notch filter exhibits a narrow transition band, significant attenuation near the central frequency, and requires no excess resistance-capacitance elements, making it well-suited for attenuating respiratory signals. Subsequently, a second amplifier circuit was employed to ensure the signal attained the ADC’s optimal input dynamic range. This circuit effectively mitigates breathing interference in BCG signal acquisition and exhibits robust anti-noise performance.

The ECG signal was not used in BCG signal processing. ECG acquisition’s purpose was solely to verify the proposed algorithm’s accuracy. The AD8232 (Analog Devices Inc., 2012) based signal conditioning block was designed for cardiac bioelectrical signals. It contains an integrated particular instrument amplifier, a suitable leg driver amplifier, an operational amplifier, and a mid-supply reference buffer. Combining a unique instrument amplifier, AD8232 can amplify the ECG signal and reject the electrode half-cell potential. The sampling circuit was designed based on the STM32F107 (STMicroelectronics, 2017) chip, with the internal analog-to-digital converter. The BCG and ECG signals are sampled synchronously in the signal conditioning circuit, with a sampling precision of 12 bits and a sampling rate of 250 Hz. The sampled data were transmitted to a host computer through a serial port. The host computer receives and processes the signals through the program developed by MATLAB.

### 2.2 Subjects and experimental procedure

Ten healthy subjects (6 males and 4 females, age: 27.1 ± 3.3 years) and two patients with CHD (male, no cardiac stenting, age: 53 and 46 years) were recruited for data collection in controlled laboratory settings. All subjects provided written informed consent before the experiment. The experiment was approved by the Ethics committee of the China Astronaut Research and Training Center.

The experiment had two stages: preparation and data collection. Initially, the subjects were instructed to lie in a bed for 3 minutes until reaching a steady resting state. Subsequently, the signal acquisition equipment previously introduced continuously recorded BCG and ECG signals with the subjects in the supine position for 10 minutes.

## 3 Methods

### 3.1 Signal pre-processing

Although a respiration separation circuit was designed for the BCG acquisition circuit, a few respiration components and high-frequency noise persist in the signal. Furthermore, the signal may experience considerable changes, surpassing the measuring range, owing to actions such as limb movements or coughing. To eliminate the residual interference signals, an FIR bandpass digital filter with a cut-off frequency of 1–20 Hz was employed. Subsequently, the signal was segmented every 10 seconds.

A digital notch filter with a center frequency of 50 Hz was used to process the ECG signal and remove the power line interference. Then, an FIR bandpass digital filter with a cut-off frequency of 1–40 Hz was used to remove the out-of-band noise, and a segmented function fit was used to remove the baseline drift. Because of the high quality of the ECG signal in the resting state, with the above processings, a simple peak detection method with a threshold was enough to locate the R wave. The R wave was used as a criterion for determining the heartbeat interval.

The BCG data was segmented and each segment lasted 10 s. 2 s were added before and after each segment to prevent the edge from corrupting. Once the heartbeat localization was completed, the heartbeat data located within these 2 s were removed.

### 3.2 IJK-complex detection algorithm

The main objective of the algorithm is to identify the IJK-complex position in the BCG signal. More specifically, it aims to locate the exact position of the three wave peaks, which are comprised of three steps. These steps will be elaborated upon in detail in the upcoming sections.

#### 3.2.1 Beat-to-beat heart rate estimation and get initial J wave location

Characteristics of the BCG signal time-domain waveform make it challenging to detect directly, and it is even difficult to distinguish each complete BCG waveform. To address this issue, Short-Time Fourier Transform (STFT) is selected as the primary method to realize beat-to-beat heart rate estimation and initial localization of J wave. The fundamental concept of the STFT is to use a time-frequency localized window function. It is assumed that g (t) is smooth (pseudo-smooth) in a relatively short time interval. By sliding g (t) and calculating the product of f (t) and g (t), the power spectrum of the signal at different moments can be derived accordingly (Wacker and Witte, 2013). The window function used in the STFT cannot be changed during the operation, and the resolution of the STFT is fixed. If the resolution needs modification, the window function must be re-chosen to achieve the desired output. This approach can ensure that a uniform resolution scale is applied to all signals. Due to the influence of the window function, the STFT exhibits local characteristics, which is a function of both frequency and time. Explicitly, the STFT is defined as:

$${{STFT}_{x}\left(t,\omega \right)=\int_{-\infty }^{\infty }x\left(\tau \right)w}^{*}\left(\tau -t\right)e^{-j\omega \tau }d\tau$$ (1)

*ω* is the analog angular frequency variable, $w\left(t\right)$ is the time window function of time-frequency localization. The commonly used time window functions include rectangular, Gaussian, Hanning, Hamming, etc.

The time scale of the time-frequency map generated by STFT is fixed, different from continuous wavelet transform (CWT). When the selected window is narrow, STFT has a higher time resolution, and STFT has a higher frequency resolution when the $w\left(t\right)$ is set wide. The characteristic of CWT is that the high-frequency part has better time resolution, while the low-frequency part has better frequency resolution. To ensure the robustness of detection, the resolution of the low-frequency region is deliberately reduced, which is the main reason for choosing the STFT method instead of CWT.

$$S\left(t\right)=\sum_{trf\left(:,4.5\right)}^{trf\left(:,7\right)}trf\left(i\right)$$ (2)

**FIGURE 1:**
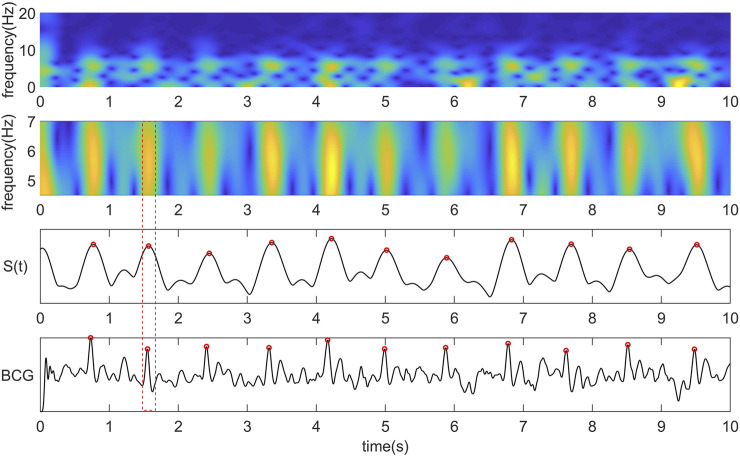
From top to bottom are the time-frequency diagram obtained by STFT, the time-frequency diagram of the most critical frequency band of BCG intercepted by us, the coefficient curve obtained by summing the time-frequency matrix of the central frequency band along the Y-axis, the original BCG signal and the corresponding relationship between the peak point of the curve and the J wave.

*trf(i)* is the coefficient matrix obtained by STFT. Curve S(t) is obtained by summing the frequency band coefficients of the J wave in the STFT coefficient matrix. $S\left(t\right)$ reflects the occurrence time of the primary energy of the BCG signal, and the peak point of the curve has a good correspondence with the position of the J wave of the BCG signal in time. Therefore, we use the time-frequency function obtained by the superposition of the main frequency band coefficients of the BCG signal to determine beat-to-beat heart rate and use it as the reference for locating each peak. The relationship between the time-frequency diagram, curve $S\left(t\right)$ and BCG signal J wave is shown in Figure 1.

**FIGURE 2:**
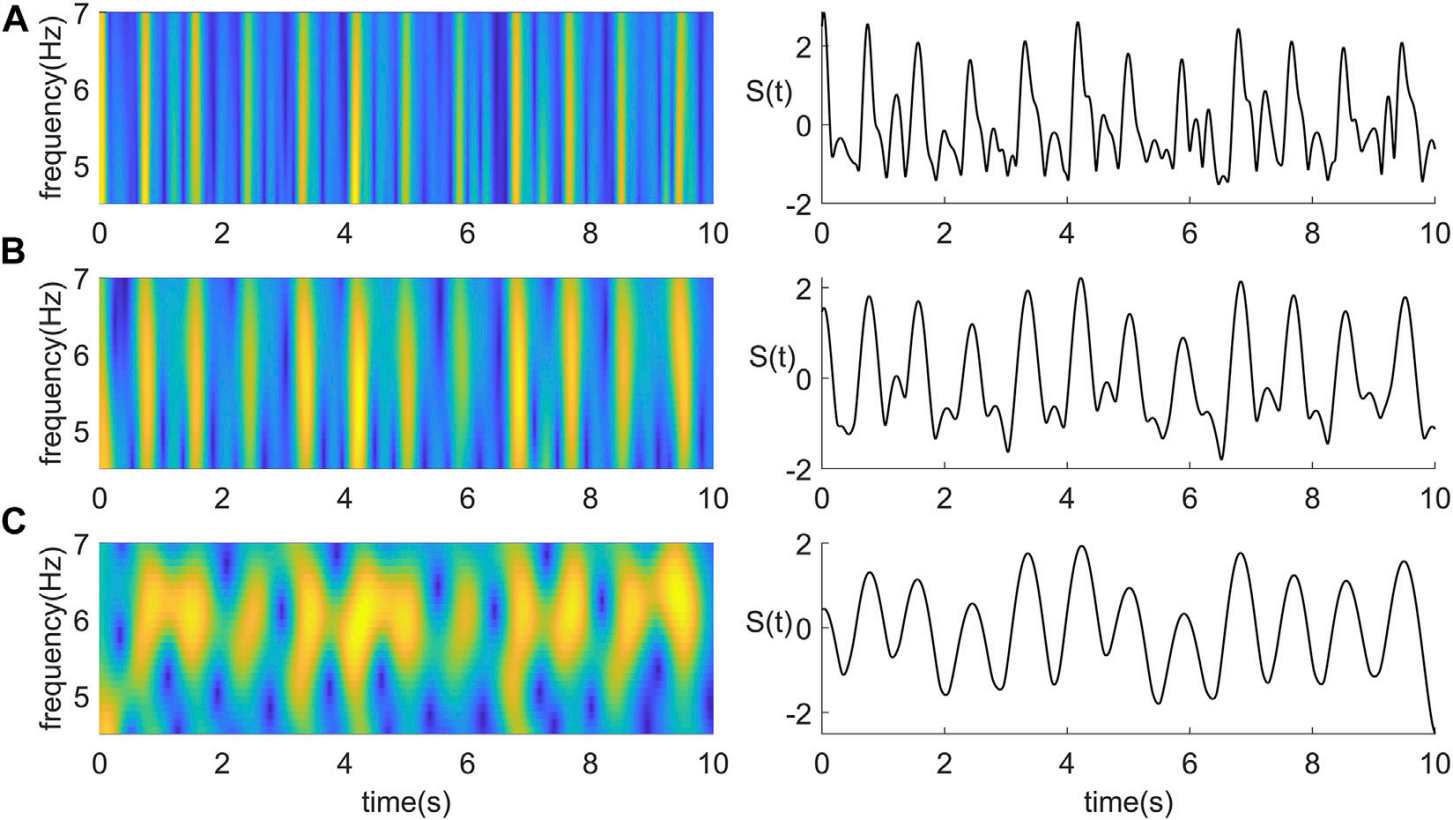
Time-frequency diagram and coefficient curve under three different frequency resolutions. **(A)** STFT using a 0.25 s Hanning window. **(B)** STFT using a 0.5 s Hanning window. **(C)** STFT using a 1 s length Hanning window.

The most critical parameter that needs to be determined is the length of the window function for STFT, which determines the frequency domain resolution. When the frequency domain resolution is higher, the coefficient fitting curve peak corresponding to the J wave is better. However, the corresponding spurious waves and other peaks of the BCG signal will become more prominent. Decreasing the frequency domain resolution can make the curve smoother, improve the accuracy of heartbeat event detection, and increase the algorithm’s robustness. However, the temporal correspondence between the peak of the curve and the J wave of the BCG will become correspondingly worse. Figure 2 shows the coefficient fittings obtained by applying three lengths of Hanning windows.

A resolution adaptive determination algorithm was designed to determine the appropriate STFT window length parameters for different individuals. First, calculate $S\left(t\right)$ with an STFT window length of $w\left(t\right)=0.3s$ , and identify all peaks of $S\left(t\right)$ . The reason why 0.3 s is chosen as the starting length is that even if severe heart rate abnormalities occur, the maximum heart rate will not exceed 200 beats per minute in a lying down resting state, with a minimum heart rate interval of 0.3 s. Then, in steps of 0.1 s, gradually increase the STFT window length until any peak value of $S\left(t\right)$ is more than 3 times larger than the other peaks in the $w\left(t\right)$ window centered at the peak point position, and determine that the $w\left(t\right)$ value is the window function length.

Increasing $w\left(t\right)$ , reducing the frequency domain resolution, and obtaining a more prominent $S\left(t\right)$ peak is to improve the robustness of heartbeat detection, which brings a loss in the temporal correspondence between the peak points of the curve and the J wave. With subsequent algorithms, it can be compensated to a certain extent.

#### 3.2.2 J wave extraction with EEMD

**FIGURE 3:**
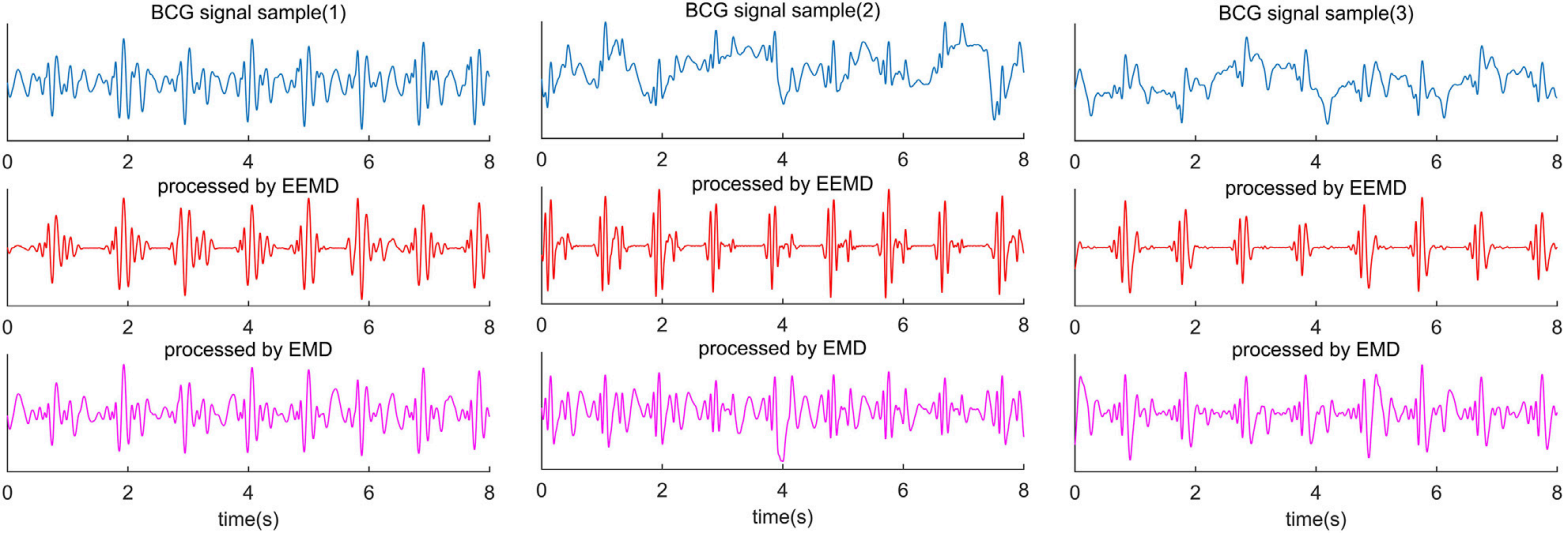
Comparison diagram of three segments of BCG signals collected from different subjects and processed by EEMD algorithm and EMD algorithm. The blue waveform in the first line is the raw BCG signal; The red waveform in the second line is the result of the EEMD algorithm, and the purple waveform in the third line is the result of the EMD algorithm.

Empirical mode decomposition (EMD) is an adaptive signal decomposition algorithm for non-linear and non-stationary signals. (Huang et al., 1998). EMD decomposes the signal based on the time scale characteristics of the data itself, without any basis function. It is essentially different from wavelet transform, Fourier transform, and other methods. The essence of EMD is to obtain the eigen wave mode through the time scale characteristic of the signal, which decomposes the signal into multiple intrinsic mode functions (IMF). Therefore, EMD can be applied to different kinds of signal decomposition without any limitations, and it has distinct advantages in non-linear and non-stationary signal processing. Despite many advantages, the algorithm has inevitable defects, such as edge effect and mode mixing problem. In particular, mode aliasing can cause the signal of one scale signal to be aliased in different IMF and even cause one IMF to lose physical significance. Modal aliasing is defined as a signal with different scales in a single IMF signal or a signal with similar scales residing in different IMF components (Djordjevich, 1981). The results of BCG decomposed by EMD are shown in Figure 3 (the last line in Figure 3 is IMF obtained by the EMD algorithm).

Ensemble empirical mode decomposition (EEMD) is an algorithm that has been developed to address the issue of mode mixing in signal decomposition. One of the key advantages of EEMD is that it introduces Gaussian white noise into the signal, thereby changing the statistical characteristics of the signal extremum points. This change in the distribution of extremum points helps to stabilize the decomposition performance of EEMD when applied to BCG signals. As a result, it becomes possible to obtain the upper and lower envelopes of the BCG signal that conform to its characteristic features. Moreover, multiple averaging techniques can be utilized to remove the added white noise and enhance the accuracy of signal decomposition using EEMD (Wu and Huang, 2011).

The EEMD algorithm incorporates two critical parameters. The first of these is the ratio between the amplitude of the white noise signal and the standard deviation of the BCG signal, denoted as Nstd. The effect of different values of Nstd on the uniformity of the distribution of extremum points varies. The second important parameter is the number of iterations of the procedure, denoted as NE. The relationship between Nstd and NE is established by Eq. 3. Generally, when the decomposition error, e ≤ 0.01, residual noise is regarded as being small enough (Wu and Huang, 2011).

$$e=\frac{Nstd}{\sqrt{NE}}$$ (3)

The Nstd is typically established based on the particular signal situation and experience, typically ranging between 1% and 5% (Wu and Huang, 2011). While some researchers have created corresponding adaptive algorithms to determine Nstd (Xu et al., 2022), such methods do not have a significant impact on BCG signal processing enhancement. Given that the noise source and frequency band of BCG signals tend to remain constant, setting Nstd to 3% can effectively enhance the distribution of extreme value points in EMD decomposition (Ni et al., 2017).

However, there is no way to identify J wave and IJK-complexes by EEMD alone accurately. The diversity of BCG signals among individuals is the biggest challenge in identifying J wave accurately. In some decomposed BCG signals, the amplitude of H and L waves reached or even exceeded the J wave amplitude (Figure 3). This is not a problem of the algorithm itself, because it happens in some subjects’ original signals. Nevertheless, 1 or 2 prominent peak points from the decomposed IMF can be easily located, and the J wave is among them. For this problem, the threshold value of the adaptive peak detection algorithm is set low. The purpose of this setup is to reduce the missed detection rate at the cost of increasing the false detection rate.

This step used half of the median of each signal segment’s eight maximum peak points after normalization as the peak detection threshold, without any other parameters qualification, such as peak distance. Such a low threshold setting can ensure effective detection.

$$\left\{Val,{LOCS}_{IMF}\left[N\right]\right\}=FindPeaks\left({BCG}_{IMF}\;{,}^{\prime }MinPeakHeight^{\prime },Threshold\right)$$ (4)

#### 3.2.3 IJK-complex detection algorithm

The underlying concept of the detection algorithm is to identify the nearest IMF peak point to the left and right of each peak point of $S\left(t\right)$ . Subsequently, one of these two IMF peak points is determined as the ultimate J wave position based on the temporal relationship and position consistency. The $S\left(t\right)$ curve is computed by summing the short-time Fourier transform coefficients of the most dominant frequency band of the BCG signal, which primarily concentrates on the J wave. Therefore, in most instances, the peak points of $S\left(t\right)$ occur proximate to the J wave. The peak point of $S\left(t\right)$ is a crucial reference for localizing J waves.

There is a critical issue to be addressed here, to strengthen the robustness of the beat-by-beat heart rate detection algorithm, we use a wider STFT window function, at the cost of diminished accuracy in J wave position. In some cases, the peak point of the J wave exhibits significant deviations from the correct position. This problem commonly arises from the high amplitude of the H wave or L wave of the BCG signal in certain individuals, causing the peak point of the J wave to shift toward the H wave or L wave. While extracting the prominent peaks in the IMF signal by EEMD, the detection threshold was relaxed to maximize the coverage of all J waves. The resultant set of peak points included several other wave peaks that required careful determination to identify the true J wave.

Previously, when extracting the major peaks in the BCG signal, the detection threshold is relaxed to maximize the coverage of all J wave. The resulting set of peak points contained many other wave peaks, in which the real J wave should be carefully determined. However, the accuracy of J wave localization is sacrificed to ensure coverage when detecting heartbeats. In some cases, the J wave’s peak point deviates significantly from the J wave. The main reason comes from the high amplitude of the H wave or L wave of the partial BCG signal in some individuals, which makes the peak point of the J wave shift toward the H wave or L wave.

**FIGURE 4:**
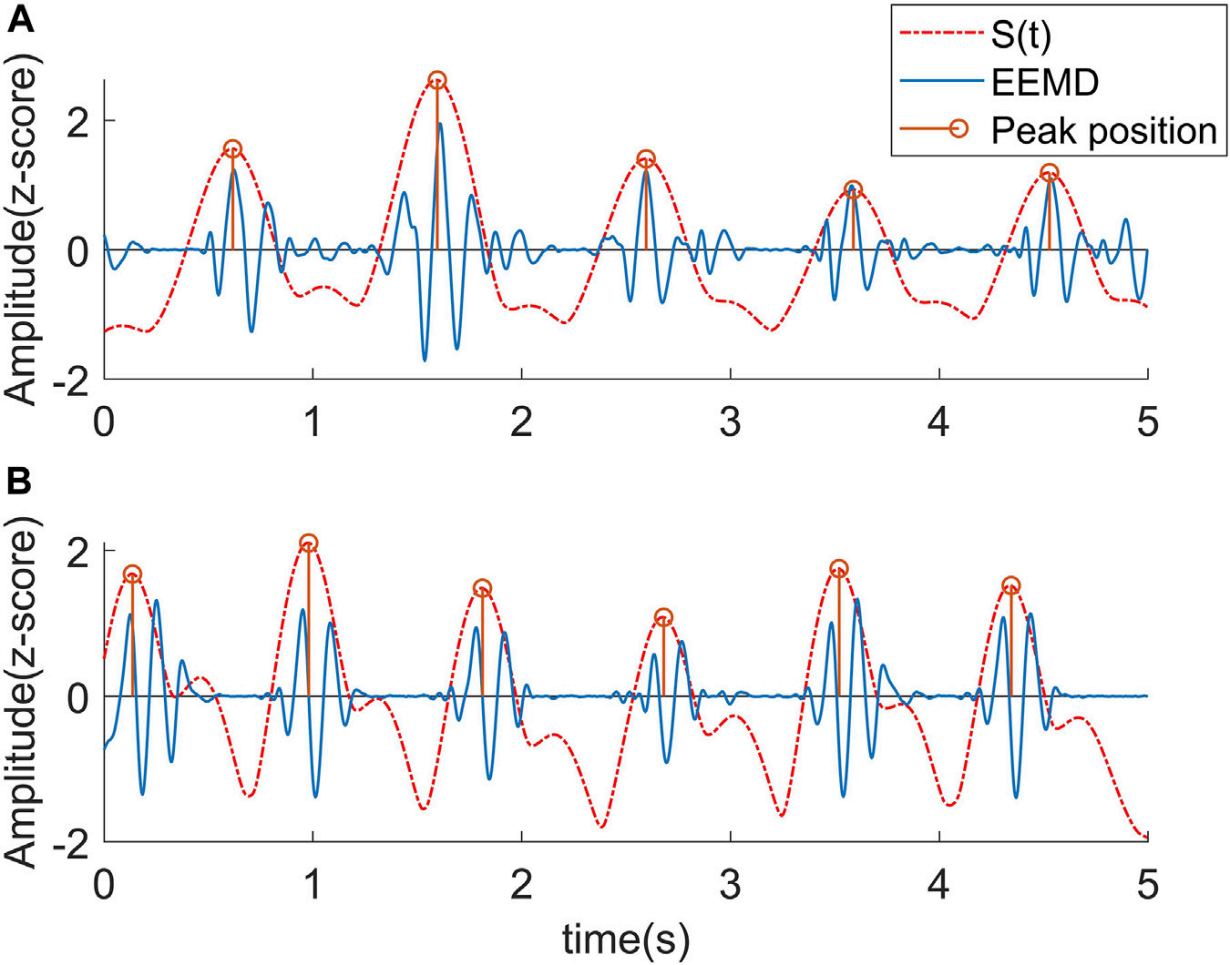
Corresponding relationship between STFT curve and J wave position. **(A)** The peaks of the curve fell near J wave. **(B)** Due to the influence of the L wave, the peaks of the curve uniformly shifted to the L wave.

The BCG signal exhibits a strong waveform consistency over a certain period of time, which confers the advantage that the overall trend of the $S\left(t\right)$ peak point shifting toward the H wave or L wave remains consistent. Specifically, the $S\left(t\right)$ peak point consistently exhibits a stable trend of peak point shifting toward the H or L wave over time (as demonstrated in Figure 4). Based on this principle, the J wave localization algorithm is presented below.

The underlying concept of the detection algorithm is to identify the nearest IMF peak point to the left and right of the peak point of $S\left(t\right)$ . The final J wave location is determined by assessing the temporal relationship and location consistency of these two points. The algorithm for identifying J waves is specified below.

The J wave identification algorithm is specified as follows (Table 1).

1) Preliminarily locating the J wave position. The peak detection algorithm with a time threshold is used in $S\left(t\right)$ , and the minimum interval is set to 0.3 s to obtain the heartbeat sequence *LOCS*_*STFT*_ [N].

$$\left\{Val,{LOCS}_{STFT}\left[N\right]\right\}=FindPeaks\left(S\left(t\right){,}^{\prime }MinPeakDistance,0.3s\right)$$ (5)

2) For each point *LOCS*_*STFT*_ [i] in the sequence of *LOCS*_*STFT*_ [N], finding 1 point in *LOCS*_*IMF*_ [N] that is closest to *LOCS*_*STFT*_ [i] on the left and right, which is noted as *LOCS*_*IMF*_ [k] and *LOCS*_*IMF*_ [k + 1].
3) If one of *LOCS*_*IMF*_ [k] and *LOCS*_*IMF*_ [k + 1] fell within the range of *LOCS*_*STFT*_ [i]±40 ms, the point is judged as the J wave position. Since the average width of the IJK-complex is about 160 ms. The closest point will be selected if both points fell within the ±40 ms range, but this situation rarely happened (occurred a total of 12 times, 0.13% of total heart beats).
4) If both *LOCS*_*IMF*_ [k] and *LOCS*_*IMF*_ [k + 1] are not within *LOCS*_*STFT*_ [i]±40 ms, it indicates that the H or L wave makes *LOCS*_*STFT*_ [i] a significant shift in position to the J wave. In this case, it would fall between the J wave and the H wave (or L wave). The relationship between *LOCS*_*STFT*_ [(i-10): (i-1)] (the 9 points before *LOCS*_*STFT*_ [i]) and the corresponding J wave need to be analyzed and the number of points that fall to the left and right of the corresponding J wave is counted. If more J waves are located to the left of *LOCS*_*STFT*_ [i] in these 9 points, then *LOCS*_*IMF*_ [k] is determined as J wave, otherwise, *LOCS*_*IMF*_ [k + 1] is determined as J wave. This is based on the principle that the tendency to shift to the H or L wave is consistent over time when the BCG signal is consistent.

**TABLE 1:** IJK-complex detection algorithm.

Algorithm IJK-complex detection
**Input:** *LOCS*_*IMF*_ [N]; *LOCS*_*STFT*_ [N]
**Output:** *LOCS*_*J*_ [N]
**for** *i* in *LOCS*_*STFT*_ [N]
find *LOCS*_*IMF*_ [*k*] and *LOCS*_*IMF*_ [*k*+1] in *LOCS*_*IMF*_ [N]
*LOCS*_*IMF*_ [*k*] = max{*LOCS*_*IMF*_ [N] < *LOCS*_*STFT*_ [*i*]}
*LOCS*_*IMF*_ [*k* + 1] = min{*LOCS*_*IMF*_ [N] > *LOCS*_*STFT*_ [*i*]}
**Switch**
**case 1** | *LOCS*_*IMF*_ [*k*]—*LOCS*_*STFT*_ [*i*] | < 40 ms *or* | *LOCS*_*IMF*_ [*k*] - *LOCS*_*STFT*_ [*i*] | < 40 ms
J wave = *LOCS*_*IMF*_ [*k or k + 1*]—*LOCS*_*STFT*_ [*i*] < 40 ms
**case 2** | *LOCS*_*IMF*_ [*k & k+1*]—*LOCS*_*STFT*_ [*i*] | > 40 ms
**If** median{*LOCS*_*IMF*_ [*k-10:k − 1*]—*LOCS*_*STFT*_ [*i − 10:k − 1*]} < 0
J wave = *LOCS*_*IMF*_ [*k*]
**else** median{*LOCS*_*IMF*_ [*k − 10:k − 1*]—*LOCS*_*STFT*_ [*i − 10:k − 1*]} > 0
J wave = *LOCS*_*IMF*_ [*k* + 1]
(Consistent with the previous 9 point offsets)
**end if**
**case 3** | *LOCS*_*IMF*_ [*k & k + 1*] - *LOCS*_*STFT*_ [*i*] | < 40 ms (Hard to happen)
J wave = *LOCS*_*IMF*_ [*k or k + 1*] near to *LOCS*_*STFT*_ [*i*]
**end switch**
**end for**

In addition, the distance between *LOCS*_*IMF*_ [k] and *LOCS*_*STFT*_ [i] is also set as a correction condition. When the selected point with the same deviation from the trend is more than twice the distance of the other point, the closest point will be chosen. In this case, the initial estimated position of the J wave has been exceeded by the more distant points by at least half the average IJK-complex wave width, or even more. This deviation from our assumptions regarding the predicted position of the J wave in the normal morphology of the BCG waveform has led us to accept values that are closer, with the aim of excluding any potential interference caused by outliers.

5) I wave and K wave is detected through J wave coordinates in IMF. I wave is the first minimum point before J wave in the IMF signal, and K wave was the first minimum point after J wave in the IMF signal. I wave and K wave also needed to meet corresponding threshold conditions, and there needed to be a zero-crossing point between I wave and J wave, and J wave and K wave.
6) It is noteworthy that while the IJK peak point’s position in the IMF exhibits good correspondence with the original signal, it demonstrates a slight deviation. Therefore, it is necessary to correct the peak detected in the IMF as well as the peak point in the original signal. The method to do so is by defining a ±3 ms time window for the IJK position located in the IMF and searching for the corresponding peak in the original signal within the window.
7) The outlier points are interpolated and replaced. The feature points within 2 s before and after the segment are removed, data is recorded, and the next segment of data calculation is started until the end.

### 3.3 Performance evaluation

The primary objective of this study is to accurately identify the IJK-complex of the BCG signal without relying on auxiliary signals such as ECG, and to further acquire the morphological characteristics and heart rate parameters of the BCG signal. To assess the algorithm’s effectiveness, a comprehensive evaluation is conducted by comparing its BCG morphological feature extraction, J wave identification, and HRV indices based on the identified J waves with the most commonly used and accurate existing methods.

Given that the error between the two methods can be either positive or negative, the mean absolute error (MAE) is utilized to measure the error of the two algorithms (Ibrahim and Bessam, 2021). The MAE is defined as follows:

$$MAE=\frac{1}{n}\sum_{i=1}^{n}\left|y_{i}-x_{i}\right|$$ (6)

$$MAE\left(\%\right)=\frac{MAE}{y_{mean}}$$ (7)

#### 3.3.1 BCG morphological feature detection

ECG-based beat gating and wave localization have emerged as the prevailing methods in prior studies for the identification of BCG waveform features (Ashouri et al., 2016; Kim et al., 2018; Shin et al., 2021a; Zhang et al., 2021). In this method, the ECG signal is initially analyzed to identify the R wave, which serves as the basis for segmenting the BCG signal into individual heartbeats. These extracted heartbeats are then averaged, and the J wave is determined as the global maximum after geometric averaging. Subsequently, the relative position between the R wave and the J wave is used to detect the J wave. A similar detection method is employed for the I-wave and K wave.

**FIGURE 5:**
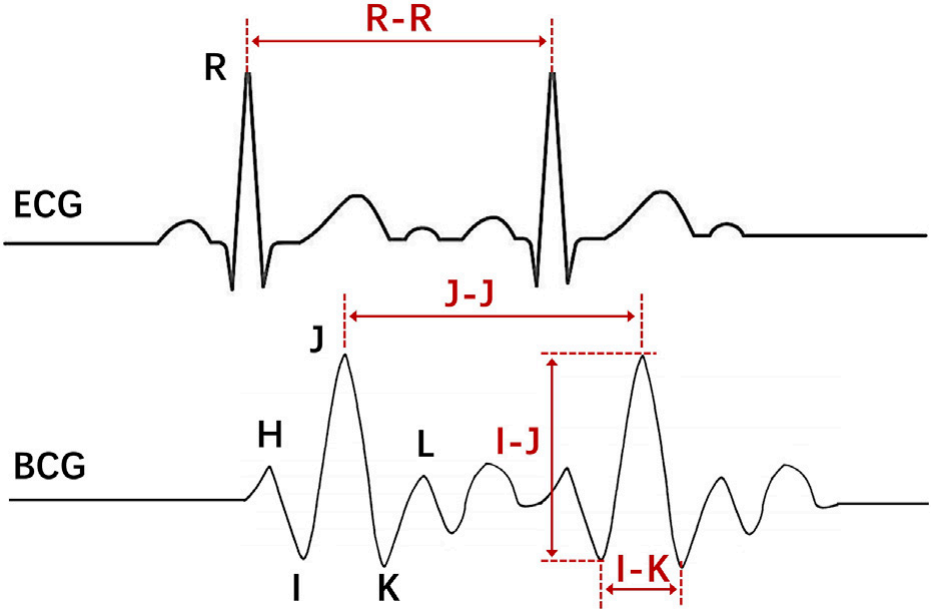
Schematic diagram of BCG and ECG signal features.

Two representative BCG morphological features, namely, the I-J amplitude and the I-K interval, have been selected for analysis (Figure 5). The I-J amplitude reflects the peak amplitude of the IJK-complex, while the I-K interval represents the width of the IJK-complex. These two indicators have been proven to be correlated with cardiac ejection function and can be utilized as effective machine learning features for automatic identification of AF (Wen et al., 2020). The two features are computed using both the ECG-assisted algorithm and the algorithm proposed in this study. MAE is used to evaluate the error between the results.

#### 3.3.2 J wave identification

At present, there is no universally accepted method available for the accurate detection of J waves in BCG signals. Furthermore, the absence of a universally accepted gold standard makes the direct quantification and assessment of J wave detection unconvincing. Consequently, we propose a comparison between the beat-to-beat heart rate estimated from J-J intervals and the R-R intervals. This approach not only reflects the accuracy of J wave recognition but also enables performance comparison with numerous existing studies that utilize BCG signal-derived IBI analysis. To facilitate comparison with prior research, the evaluation of heart rate estimation errors also incorporates the root mean square error (RMSE). The RMSE is defined as follows:

$$RMSE=\sqrt{\frac{\sum_{i=1}^{n}{\left(y_{i}-x_{i}\right)}^{2}}{n}}$$ (8)

In addition, the mean deviation and the standard deviation of the difference between the two data sets are computed. If the difference obeys a normal distribution, 95% of the deviation values will lie within the mean ±1.96SD limits. Finally, Bland-Altman plots for each subject’s data are drew to assess whether there is a systematic bias in the results obtained by the two algorithms (Bland and Altman, 2010).

#### 3.3.3 HRV estimation

Heart rate variability (HRV) refers to the minor differences in instantaneous heart rate between consecutive heartbeats or the pattern of change in the beat-to-beat interval (Tiwari et al., 2021). HRV is an important method for the analysis of cardiac activity. The features extracted by time and frequency domain methods are well defined and easily understood. It is commonly used in clinical cardiovascular disease diagnosis and monitoring. In this paper, eight most commonly used time and frequency domain HRV indicators are selected. They are calculated using the R-R sequence of ECG and the J-J sequence of BCG signal extracted by presented algorithm. *N* in the following equation represents the number of all samples, and RRi (JJi) represents the time difference between adjacent R waves (J waves).

Heart rate interval mean, RRMean (JJMean):

$$RRMean=\frac{1}{n}\sum_{i=1}^{n}{RR}_{i}$$ (9)

R-R (J-J) interval standard deviation, SDNN:

$$SDNN=\sqrt{\frac{\sum_{i=1}^{n}{\left({RR}_{i}-RRMean\right)}^{2}}{n}}$$ (10)

PNN50 indicates the number of times that the RR (JJ) interval difference is greater than 50 ms.

RR (JJ) interval difference root mean square, MSSD:

$$MSSD=\sqrt{\frac{\sum_{i=1}^{n-1}{\left({RR}_{i+1}-{RR}_{i}\right)}^{2}}{n-1}}$$ (11)

The coefficient of variation (CV) is defined as the ratio of the standard deviation to the mean of the RR (JJ) interval:

$$CV=\frac{SDNN}{RRMean}$$ (12)

The frequency-domain analysis method needs a selection of a signal segment (usually greater than 256 heartbeat points) for spectral analysis. Studies divided the HRV spectrum into three frequency bands, very low frequency (VLF, <0.04 Hz), low frequency (LF, 0.04–0.15 Hz), and high frequency (HF, 0.15–0.4 Hz). In this study, low-frequency power (LF), high frequency (HF), and total power (TP) are chosen.

Paired samples *t*-test were used to assess the statistically significant differences between the indicators of the eight HRV characteristics obtained by BCG and ECG. Furthermore, the consistency of the results obtained by the two algorithms was assessed by Bland-Altman plots.

## 4 Results

### 4.1 BCG morphological feature detection

Table 2 shows the I-K interval and I-J amplitude outcomes obtained using the algorithm presented in this study and the ECG-based IJK-complex localization algorithm. The algorithm exhibited a notable performance in the estimation of the I-K interval, with an average MAE of 1.48 ms. The minimum MAE was only 0.2 ms and the maximum was 6.27 ms. The MAE of two patients with CHD were 0.2 ms and 2.15 ms, maintaining the same level of error as other healthy subjects.

**TABLE 2:** I-K interval and I-J amplitude estimated accuracy.

Subject	I-K interval (M (SD), ms)	MAE (ms)	MAE (%)	I-J amplitude (M (SD), V)	MAE (V)	MAE (%)
1	171.63 (4.45)	1.41	0.82	2.96 (0.68)	0.05	1.75
2	192.00 (9.81)	6.27	3.27	3.74 (0.77)	0.04	1.07
3	139.08 (8.15)	2.57	1.85	5.43 (0.46)	0.01	0.19
4	150.48 (4.11)	0.81	0.54	4.08 (0.72)	0.05	1.26
5	152.24 (3.58)	0.23	0.15	4.56 (0.49)	0.01	0.27
6	170.03 (4.70)	0.90	0.53	4.87 (1.44)	0.20	4.11
7	191.30 (2.13)	0.59	0.31	3.19 (0.39)	0.06	1.92
8	183.72 (2.83)	0.59	0.32	3.98 (0.65)	0.03	0.85
9	159.92 (3.48)	0.32	0.20	4.50 (0.54)	0.01	0.27
10	163.78 (5.61)	1.73	1.06	4.23 (1.00)	0.06	1.39
*11	175.31 (5.79)	2.15	1.22	3.17 (0.77)	0.12	3.79
*12	166.24 (3.36)	0.20	0.12	4.75 (1.06)	0.01	0.21
mean	167.98 (4.83)	1.48	0.87	4.12 (0.75)	0.05	1.42

* Patients with CHD.

**FIGURE 6:**
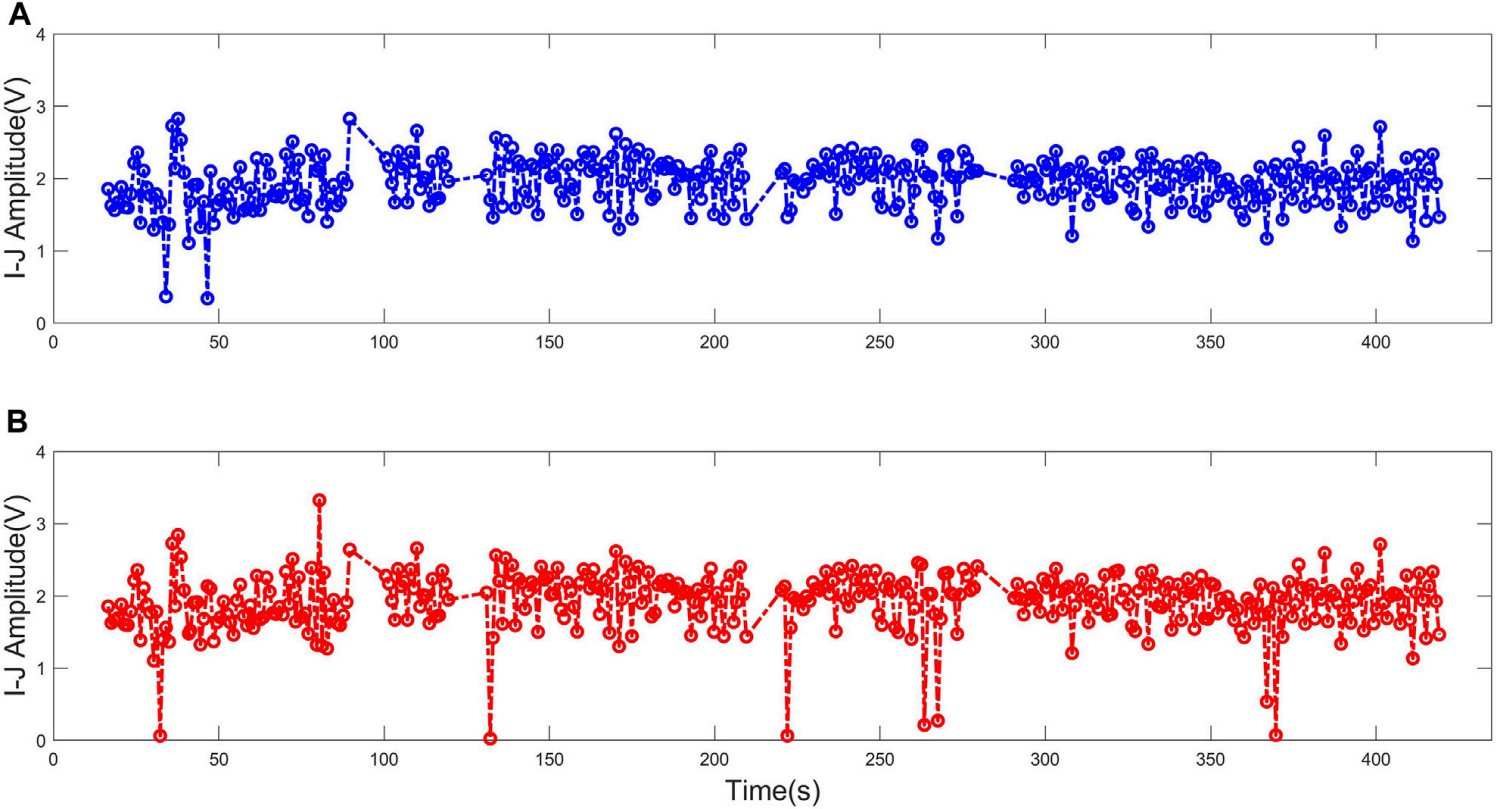
The comparison between the I-J amplitude data obtained utilizing two methods. **(A)** I-J amplitude obtained utilizing ECG-based algorithm. **(B)** I-J amplitude obtained utilizing the presented algorithm.

Regarding the I-J amplitude, we observed a MAE of 0.05 V. Moreover, the MAE indicated no significant performance differences between the two CHD patients and healthy subjects. Figure 6 illustrates the I-J amplitude data obtained from both methods. Overall, a strong consistency can be observed between the two methods, despite the presence of significant deviations at individual points.

### 4.2 J wave identification (heart rate estimation)

**FIGURE 7:**
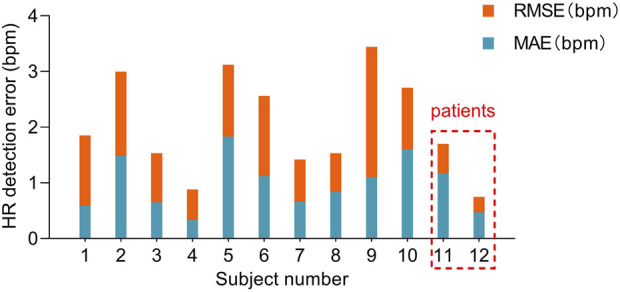
MAE and RMSE of heart rate estimated from the presented algorithm and the heart rate measured from ECG. Subjects No. 11 and No. 12 were patients with CHD.

**FIGURE 8:**
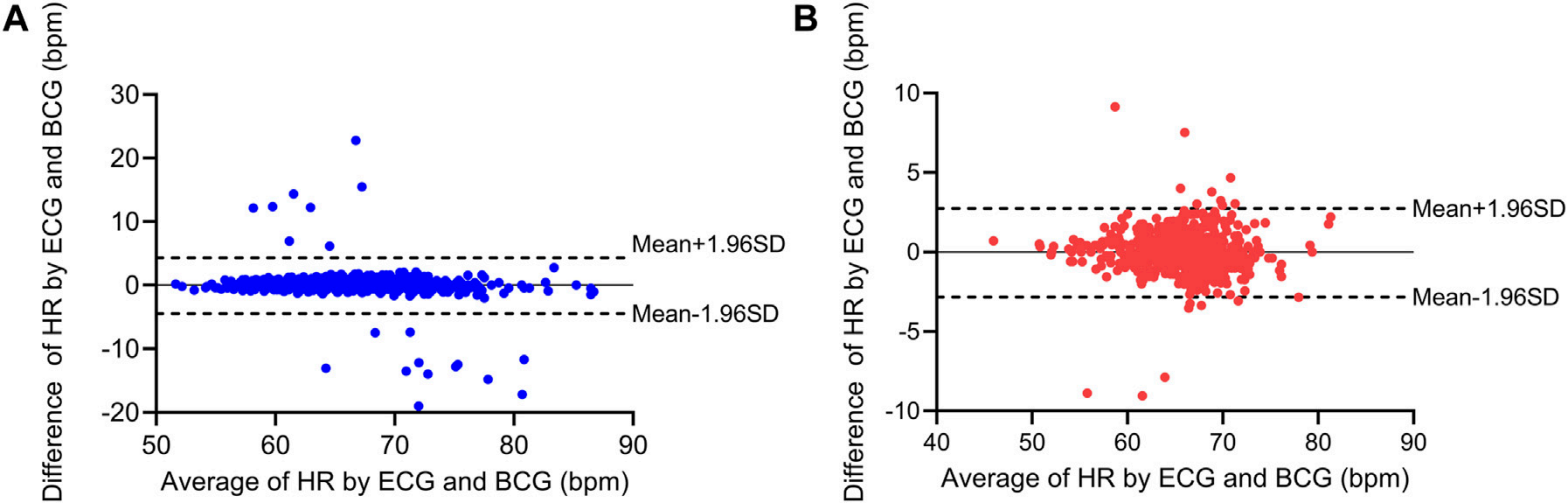
Bland-Altman plots for the heart rate estimation of two subjects. **(A)** Bland-Altman plots for subject No. 1 (healthy subject). **(B)** Bland-Altman plots for subject No. 12 (patients with CHD).

Based on the detection and assessment methods presents above, Beat-to-beat heart rate estimation was performed on 12 subjects, and the results are summarized in Table 3; Figure 7. The average heart rate estimated from the R-R interval of ECG was 68.23 bpm and 68.08 bpm from the J-J interval of BCG. The maximum deviation in mean heart rate for all subjects was 0.15 bpm. The MAE of heart rate for all subjects was 0.99 bpm with a 2.80 bpm 95% confidence interval. The maximum MAE was 1.83 bpm and the minimum was 0.33 bpm. The RMSE, which is more sensitive to significant errors due to its higher norm, was 2.04 bpm, with the maximum value being 3.44 bpm, and the minimum value was 0.75 bpm. The Bland-Altman plot, a suitable approach for comparing two medical measurement methods, showed that the variation in the Beat-to-Beat heart rate among the 12 subjects ranged from −0.08 to 0.6 bpm, and the 95% confidence interval (±1.96 SD) ranged from ±1.47 to ±6.74 bpm. The Bland-Altman plots of subject NO. 1 and subject NO. 12 are shown in Figure 8. It is worth noting that subjects No. 11 and No. 12 in the table are patients with CHD, and the MAE of heart rate estimations is 1.17 bpm and 0.47 bpm. The algorithm did not show significant performance degradation on them.

**TABLE 3:** Accuracy of heart rate estimation [MAE (bpm), RMSE (bpm)].

Subject	HR (M (SD))	MAE	MAE (%)	RMSE	Coverage (%)
1	72.94 (4.72)	0.59	0.81	1.85	95.88
2	81.18 (4.79)	1.49	1.84	3.00	97.44
3	59.73 (2.71)	0.65	1.09	1.53	95.56
4	59.16 (2.23)	0.33	0.56	0.88	95.44
5	62.49 (4.18)	1.83	2.93	3.12	89.89
6	63.39 (4.01)	1.12	1.77	2.56	95.85
7	68.81 (2.59)	0.66	0.96	1.42	89.09
8	63.80 (2.88)	0.84	1.32	1.53	95.66
9	74.60 (6.91)	1.10	1.47	3.44	92.97
10	65.30 (4.34)	1.60	2.45	2.71	91.40
*11	85.55 (2.06)	1.17	1.37	1.70	91.79
*12	59.97 (2.06)	0.47	0.78	0.75	93.34
Mean	68.08 (3.62)	0.99	1.45	2.04	93.69

* Patients with CHD.

### 4.3 HRV estimation

**FIGURE 9:**
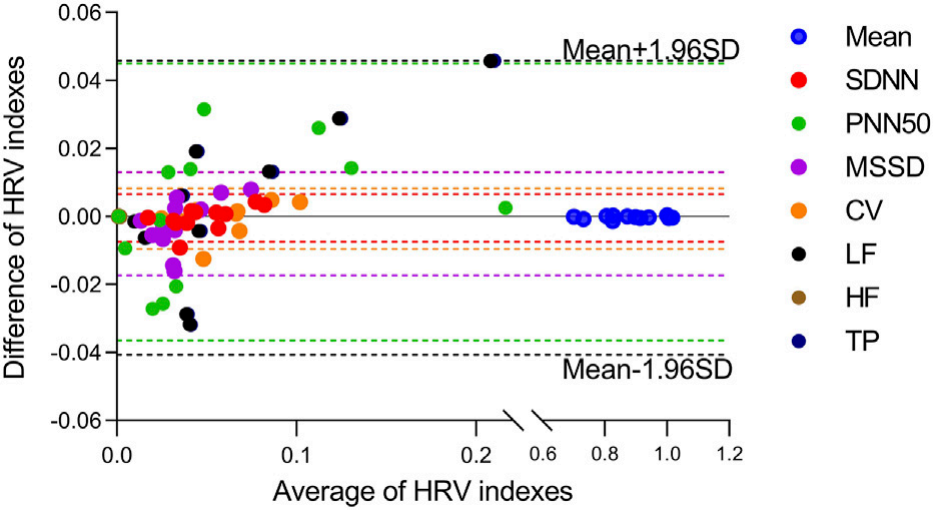
Bland-Altmann plots for eight HRV indicators estimated from the presented algorithm and the heart rate measured from ECG. Indexes were marked with different colors.

The HRV characteristics were computed from the R-R interval of the ECG and the J-J interval of the BCG sequence. The Bland-Altman plots (Figure 9) for the eight indicators obtained from both measuring methods demonstrated that the error values between the two methods were within the Mean ±1.96 SD range. In order to assess whether the HRV characteristics acquired from the two measurement approaches were significantly different, a paired-sample *t*-test was conducted on each of the eight HRV characteristics for each participant, and the *p*-values were computed. The results are displayed in Table 4. There was no significant difference (*p* > 0.05) in any of the HRV characteristics acquired by the two methods.

**TABLE 4:** HRV indicators statistics results.

HRV	ECG (M±SD)	BCG (M±SD)	Mean absolute error	t value	*p*-value
mean	0.878 ± 0.105	0.878 ± 0.104	0.000280	−2.014	0.069
SDNN	0.048 ± 0.020	0.048 ± 0.018	0.000440	−0.429	0.676
PNN50	0.058 ± 0.068	0.056 ± 0.061	0.001464	0.262	0.798
MSSD	0.034 ± 0.019	0.037 ± 0.015	0.002154	−0.962	0.357
CV	0.054 ± 0.024	0.055 ± 0.022	0.000644	−0.491	0.633
LF	0.060 ± 0.065	0.057 ± 0.049	0.002641	0.415	0.686
HF	0.001 ± 0.000	0.001 ± 0.000	0.000013	0.411	0.689
TP	0.061 ± 0.066	0.058 ± 0.049	0.002654	0.416	0.685

## 5 Discussion

The present study aimed to propose an algorithm for the identification of the IJK-complex in BCG signals. The main feature of the algorithm is its capability to detect the specific locations of the IJK-complex in BCG signals, thereby offering more valuable information for the research and application of BCG. This significant distinction sets it apart from traditional methods that primarily focus on the estimation of IBI.

Firstly, the detection of the IJK-complex enables the acquisition of diverse features pertaining to the amplitude, width, and waveform morphology of the peaks in the BCG signal. The morphological features of the BCG signal hold significant value in assessing cardiac ejection, CO, detecting atrial fibrillation (Brueser et al., 2013b; Jiang et al., 2021b), and monitoring heart failure patients (Zhang et al., 2021).

I-J amplitude is a parameter highly associated with CO. It has been demonstrated in studies that while there is substantial variation in the shape and amplitude of the BCG waveform among individuals, the variability within the same individual is comparatively low (Etemadi and Inan, 2018). Hence, the relative changes in CO can be robustly derived from the temporal measurements of the morphological features of BCG signal within an individual. The I-J amplitude measured by the proposed algorithm exhibits a comparatively small error (MAE of 1.42%) in comparison to the ECG-based method. As illustrated in Figure 6, the primary source of error between the proposed method and the ECG-based method is not systematic bias, but rather outlier values resulting from the inaccurate detection of individual points. These outlier values can be readily interpolated and replaced using algorithms without compromising the overall utilization of the features.

The morphological features of the BCG signal are closely associated with the activity of the cardiovascular system and arterial pressure, as dictated by the generation mechanism of BCG (Guidoboni et al., 2019). A wider range of possibilities for establishing BCG morphological features and clinical alterations is offered by machine learning methods. Atrial fibrillation, the most prevalent cardiac arrhythmia, often gives rise to various heart conditions like stroke and heart failure. machine learning models can enable atrial fibrillation detection (Wen et al., 2020) by utilizing morphological features such as the amplitude and interval of the BCG signal, as well as the skewness and kurtosis of the IJK-complex.

Secondly, the detection of the IJK-complex allows us to obtain the specific location of the J wave rather than just the IBI. An important application scenario for identifying the specific location of the J wave involves the measurement of pulse transit time (PPT). In a recent study, the utilization of BCG signals, instead of ECG signals, was employed to determine PPT for the detection of cuffless blood pressure tracking (Shin et al., 2021b). This approach liberates blood pressure monitoring from the constraints of ECG electrodes, thereby facilitating a more comfortable and convenient method for tracking and monitoring blood pressure. Such monitoring can be effortlessly achieved through the utilization of portable devices such as bracelets and mattresses.

Thirdly, the IBI and heart rate can be easily obtained through the identification of the IJK-complex. Due to the absence of a recognized gold standard for J wave detection in BCG signals, the primary objective of our IBI study was to substantiate the validity of J wave detection. Jung et al. used the Probability Density Function and fused multi-channel data to achieve a MAE of 1.78 bpm in the supine position, based on the data collected from eleven healthy subjects (Jung et al., 2021). Jiao et al. reported a remarkable performance of the LSTM network with the lowest MAE of 0.68 (Jiao et al., 2021). However, it should be noted that Jiao’s MAE of heart rate was computed and averaged every 15 s on the test data. Another study employed the commonly used CWT method to process BCG signals and obtained a mean error of −0.03 bpm (not MAE) and a 95% confidence interval of ±2.7 bpm for heart rate estimation, based on the results obtained from seven healthy subjects (Alvarado-Serrano et al., 2016). The proposed algorithm exhibits a heart rate estimation capability comparable to the aforementioned methods, with an MAE of 0.99 bpm and a 95% confidence interval of ±2.8 bpm. By comparing our algorithm to previous studies that estimated heart rate based on IBI, we found that the error in heart rate obtained through J wave detection is comparable. This observation confirms the accuracy of our algorithm in detecting the position of the IJK-complex.

Furthermore, a noteworthy capability in estimating HRV is exhibited by our algorithm, thereby enhancing the feature system for non-invasive cardiac function monitoring. The HRV indices obtained from BCG and ECG are inherently biased due to the variability of individual R-J intervals. One of the key factors is associated with cardiac ejection and systolic blood pressure (Deliere et al., 2013). A study conducted on 92 healthy subjects showed that R-J intervals measured by a weighing scale system ranged from 203-290 ms (Inan, 2009). Another study revealed that the R-J interval had a standard deviation of 20 ms (Etemadi et al., 2011). However, our study showed that there was no statistically significant difference in HRV obtained by BCG and ECG. Among the eight HRV features, the temporal domain features exhibited better consistency than the frequency domain features, with the exception of PNN50. This outcome is consistent with our initial expectations. PNN50 registers the number of instances when the RR (JJ) interval difference is greater than 50 ms, which is more susceptible to the influence of inherent R-J intervals and false detection points, as well as frequency-domain characteristics. HRV analysis based on BCG signaling has potential applications in portable sleep testing and heart disease detection.

Coverage, as a crucial metric for BCG signal analysis, is of paramount importance. It is undesirable to exclude a significant portion of the data in order to minimize error. In our study, the coverage ranged from 89.09% to 97.44%. The majority of the uncovered data can be attributed to motion artifacts, which resulted in distorted data. Currently, there is no existing method to accurately detect heart rate from such corrupted signals. In future endeavors, we anticipate further enhancements to improve the algorithm’s coverage. One potential approach to achieve this goal is through the utilization of a multi-channel fusion algorithm, as proposed by Jung et al (Jung et al., 2021).

Finally, a brief comparison is made between our proposed method and the main existing BCG processing methods. Methods such as template matching, autocorrelation function, cepstrum analysis, fast Fourier transform, and continuous wavelet transform (CWT) have been used to estimate IBI rather than locating the J wave (Ibrahim and Bessam, 2021). For instance, the template matching algorithm identifies the position of the heartbeat on the correlation coefficient map of the original signal and template (Paalasmaa et al., 2015). Similarly, the autocorrelation function method searches for IBI on the adaptive window autocorrelation function. The CWT method, which is a time-frequency transform that extends the short-time Fourier transform, is widely used in time-frequency analysis. This method involves estimating the correlation between the signal and different scales of wavelets, and plotting the variation in correlation for each wavelet over a specific period. These aforementioned methods all share the limitation of obscuring the morphological information of the BCG waveform in order to estimate IBI. Both the correlation coefficient map and the wavelet coefficient curve fail to incorporate the morphological information of the BCG waveform. Moreover, the extremal points on the coefficient curve do not correspond to specific locations within the BCG waveform. Another method commonly used for estimating heart rate involves employing DWT and EMD algorithms to generate a wavelet coefficient curve or IMF (Ibrahim and Bessam, 2021). DWT and EMD-based algorithms can also be utilized to remove out-of-band noise from signals and facilitate the detection of the J wave. In the DWT approach, the BCG signal undergoes multiresolution analysis by summing several detailed components to reconstruct the cardiac signal. The EMD algorithm necessitates the summation of several IMFs for cardiac signal reconstruction. Despite both methods exhibiting better noise removal capability, a time-domain peak detection algorithm must be implemented to detect J peaks. However, the robustness of J-peak detection cannot be effectively guaranteed due to the absence of other reference standards.

The algorithm introduced in this work is capable of determining the precise position of the J wave rather than just the IBI, preserving the IJK position feature of the original signal. The algorithm uses a short-time Fourier transform and summation across frequencies to obtain an initial estimate of the J point occurrence using peak finding, followed by EEMD and a regional search to precisely identify the J point. Given that the resolution of the frequency domain can be adjusted as needed with the use of STFT, its role in producing reliable results cannot be understated. In our study, we have moderately reduced the frequency band resolution to better match the peaks and J waves, which can help lower the likelihood of missed or false detections and improve the overall robustness of the algorithm. Nevertheless, this reduction of resolution can increase the deviation between the frequency curve peak and the J wave position. To address this issue, we have applied the EEMD algorithm to correct these deviations. The presence of noise in the frequency band of the BCG signal is the primary factor that affects the quality of the signal. Furthermore, these noises are challenging to eliminate through filters. Notably, these noises overlap with the frequency band of the BCG signal but do not possess repeated waveforms. To address this challenge, the EEMD algorithm is utilized, which relies on the intrinsic modal properties of the signal. This algorithm can effectively segregate the noise and the BCG signal into different IMFs. The IMF containing the BCG signal retains the original positional features of the IJK-complex. Based on an analysis of the reasons why the peak deviates in STFT, we propose a hypothesis that the deviation direction of the prominent peak is consistent. We have conducted statistical analysis on 12 human subjects and observed that the deviation trend for 98.87% of the points with deviations longer than 40 ms is consistent. This observation suggests that the hypothesis is valid, particularly in relatively short time ranges. By utilizing this approach, we can combine the benefits of both algorithms to enhance our ability to calibrate the J wave position in the IMF.

The study possesses several limitations that warrant acknowledgment. Firstly, the BCG waveform can be distorted or entirely submerged in motion artifacts, rendering it undetectable. Additionally, body movements can lead to loss of the original waveform. Secondly, suboptimal outcomes are observed when extracting the L wave, M wave, and N wave using the EEMD algorithm. Many individuals exhibit small amplitudes for these waves in BCG signals. Moreover, the repeatability of the M wave and N wave is relatively poor. Subsequent to EEMD and multiple averaging, these waves nearly vanish, presenting challenges in their effective identification within the IMFs. Thirdly, further comprehensive investigations are required to assess the algorithm’s suitability for long-term monitoring and patients with cardiovascular diseases. Given the aforementioned issues, we are presently developing enhanced acquisition hardware, data recording, and signal processing systems to facilitate the adoption of this non-invasive method in various medical applications.

## 6 Conclusion

There is a trade-off between maximizing beat-to-beat heart rate estimation accuracy and preserving the original BCG waveform information. Most existing methods improve heart rate estimation accuracy by sacrificing BCG waveform features. To address this limitation, this paper proposes a novel BCG feature detection algorithm that combines STFT and EEMD. By leveraging the advantages of both algorithms, the proposed method achieves high level heart rate estimation and precise localization of the IJK wave in BCG. The proposed method enhances the BCG morphological feature system and expands the available indicators for subsequent studies of non-invasive sleep monitoring, predicting atrial fibrillation, and cardiac function monitoring.

## Data Availability

The original contributions presented in the study are included in the article/Supplementary material, further inquiries can be directed to the corresponding author.
